# Protein tyrosine phosphatase PTPN1 modulates cell growth and associates with poor outcome in human neuroblastoma

**DOI:** 10.1186/s13000-019-0919-9

**Published:** 2019-12-14

**Authors:** Caroline E. Nunes-Xavier, Olaia Aurtenetxe, Laura Zaldumbide, Ricardo López-Almaraz, Asier Erramuzpe, Jesús M. Cortés, José I. López, Rafael Pulido

**Affiliations:** 1 Biomarkers in Cancer Unit, Biocruces Bizkaia Health Research Institute, Barakaldo, Bizkaia Spain; 2 Department of Tumor Biology, Institute for Cancer Research, Oslo University Hospital Radiumhospitalet, P.O. Box 4950 Nydalen, N-0424 Oslo, Norway; 3 Department of Pathology, Cruces University Hospital, University of the Basque Country (UPV/EHU), Barakaldo, Bizkaia Spain; 4 Pediatric Oncology and Hematology, Cruces University Hospital, Barakaldo, Bizkaia Spain; 5 Quantitative Biomedicine Unit, Biocruces Bizkaia Health Research Institute, Barakaldo, Bizkaia Spain; 6 IKERBASQUE, Basque Foundation for Science, Bilbao, Spain; 7 Biocruces Bizkaia Health Research Institute, Hospital Universitario de Cruces, Plaza Cruces s/n, 48903 Barakaldo, Spain

**Keywords:** Neuroblastoma, Neuroblastoma differentiation, Metastatic neuroblastoma, Protein tyrosine phosphatases, PTPN1

## Abstract

**Background:**

Protein tyrosine phosphatases (PTPs) regulate neuronal differentiation and survival, but their expression patterns and functions in human neuroblastoma (NB) are scarcely known. Here, we have investigated the function and expression of the non-receptor PTPN1 on human NB cell lines and human NB tumor samples.

**Material/methods:**

NB tumor samples from 44 patients were analysed by immunohistochemistry using specific antibodies against PTPN1, PTPRH, PTPRZ1, and PTEN. PTPN1 knock-down, cell proliferation and tyrosine phosphorylation analyses, and RT-qPCR mRNA expression was assessed on SH-SY5Y, SMS-KCNR, and IMR-32 human NB cell lines.

**Results:**

Knock-down of PTPN1 in SH-SY5Y NB cells resulted in increased tyrosine phosphorylation and cell proliferation. Retinoic acid-mediated differentiation of NB cell lines did not affect PTPN1 mRNA expression, as compared with other PTPs. Importantly, PTPN1 displayed high expression on NB tumors in association with metastasis and poor prognosis.

**Conclusions:**

Our results identify PTPN1 as a candidate regulator of NB cell growth and a potential NB prognostic biomarker.

## Background

Neuroblastoma (NB) constitutes a heterogeneous malignancy arising from sympathetic neural precursor cells from the neural crest. NB is the most common extracranial tumor in children, accounting in its high-risk forms for about 15% of childhood cancer mortality. The majority of NB tumors develop in the adrenal medulla, and, with less frequency, in the paraspinal ganglia from neck, chest, abdomen, and pelvis [1–4]. Unbalanced neuronal differentiation seems to be an important aspect in NB initiation and progression, with many primary and metastatic NB tumors showing spontaneous regression [5, 6]. High-risk NB do not show regression and are currently treated with a combination of chemo- and radio-therapy protocols, autologous stem cell transplant, and surgery, followed by maintenance therapies based on immuno-therapy and retinoic acid (RA) cell differentiating agents. However, high-risk NB are highly malignant and frequently relapse after these combination therapies, rendering a survival lower than 50% of treated children [7, 8]. Improvements in the definition of therapy response biomarkers and in the implementation of more effective targeted therapies are current necessities in the management of high-risk NB patients [9–12].

Tyrosine phosphorylation, executed by protein tyrosine kinases (PTKs), is one of the landmark protein posttranslational modifications in human cancer [13]. The receptor tyrosine kinase (RTK) ALK is mutated in NB tumors and in the germline of patients with familial NB [14–16], and ALK plays a major role in the signal transduction mechanism driving NB development, being a major potential target in NB targeted therapy [17, 18]. ALK hyperactivation associates in NB with *MYCN* gene amplification, the major biomarker for high-risk NB, and potentiates MYCN oncogenic activity [19, 20]. In addition, the RTKs TrkA and TrkB are major signalling transducers in the regulation of NB growth, differentiation, and apoptosis, whose expression has been correlated with NB prognosis and regression, and whose therapeutic targeting is also being addressed in NB [21, 22]. These examples illustrate that alterations in the tyrosine phosphorylation cellular status of NB cells are crucial for NB development and progression. However, the regulated protein tyrosine dephosphorylation in NB has been scarcely investigated.

Protein tyrosine phosphatases (PTPs) are the direct executers of dephosphorylation of specific tyrosine residues on specific protein substrates, playing relevant roles in many physiologic and pathologic processes, including those related with the regulation of cell differentiation, transformation, and growth [23–27]. Inhibition of PTPs by vanadium compounds enhances RA-triggered differentiation of NB cells, suggesting an active role for these enzymes in NB cell proliferation and senescence [28]. Moreover, PTP inhibition by vanadate or vanadium compounds courses with induced cell death on NB cell lines, making PTP inhibition a suitable therapeutic option in NB [29]. The non-receptor tyrosine phosphatase PTPN1 (also known as PTP1B) constitutes the paradigm of PTP enzymes and a suitable drug target for cancer and metabolic diseases [30–33], and PTPN1 protein expression has been shown to correlate with metastasis and poor prognosis in several human cancers [34–37]. In this study, analysing human NB tumor samples and cell lines, we have found evidence for PTPN1 as a regulator of NB cell tyrosine phosphorylation and proliferation, and unveiled the association of PTPN1 expression with poor NB patient outcome.

## Methods

### Patients, tissue specimens, and immunohistochemistry

The characteristics of the patients included in the study have been previously described [38] (Table 1). Histological sections of tissue microarrays (TMAs) or routine paraffin blocks containing the tumor specimens were used for immunohistochemistry (IHC). The antibodies and dilutions used for IHC were: PTEN (1/50, 6H2.1, Merck Millipore), PTPRZ1 (1/50, Clone 12/RPTPb, BD Bioscience), PTPRH (1/500, HPA042300, Sigma-Aldrich), and PTPN1 (1/20, AF1366, R&D). Immunostainings were performed in automated immunostainers (EnVision FLEX, DakoAutostainer Plus; Dako, Glostrup, Denmark) following routine methods. The analysis was done blind by an experienced pathologist (LZ) and performed using a Nikon Eclipse 80i microscope (Tokyo, Japan). The IHC evaluation considered positive (high) those cases with intense nuclear or granular cytoplasmic staining positive cells, and negative (low/no) those with weak or non-existent staining positive cells). Each examined core and routine paraffin block contained a minimum of 200 tumor cells.

**Table 1:** Clinic-pathologic characteristics and *MYCN* amplification of study population

Patients (*n* = 44)	%	MYCN	
		amp	No-amp
Gender		(*p* = 0.604) (*R* = -0.090)	
Male (21)	48	3	12
Female (23)	52	5	13
Age at diagnosis (months)		**(*p*** **= 0.023)** (*R* = 0.396)	
< 18 (29)	66	3	20
> 18 (15)	34	5	5
Risk		**(*p*** **= 0.003)** (*R* = 0.516)	
Intermediate, high (23)	52	8	10
Low (21)	48	8	0
Stage		**(*p*** **= 0.002)** (*R* = 0.550)	
Metastatic (12)	27	6	4
Non-metastatic (32)	73	2	21
Stage		**(*p*** **= 0.018)** (*R* = 0.412)	
III, IV (17)	39	6	7
non-III, non-IV (27)	61	2	18
Stage		**(*p*** **= 0.000)** (*R* = 0.606)	
IV (13)	30	6	3
non-IV (31)	70	2	22
Survival		**(*p*** **= 0.000)** (*R* = 0.650)	
Dead (10)	23	5	1
Alive (34)	77	3	24

Note that information on *MYCN* amplification is not available for all samples

Significant *p* values (*p* < 0.05) are in bold

### Cell lines and reagents

Human NB cell lines SH-SY5Y (ALK F1174 L), SMS-KCNR (ALK R1275Q, *MYCN* amplified), and IMR-32 (*MYCN* amplified) are from ATCC. Cells were grown at 37 °C in a humidified 5% CO2, 95% air incubator. SH-SY5Y and IMR-32 cells were grown in DMEM/F12 supplemented with 10% FBS, 2 mM L-glutamine, 100 units/ml of penicillin, 0.1 mg/ml of streptomycin, and 1% non-essential amino acids. SMS-KCNR were grown in DMEM supplemented with 10% FBS, 2 mM L-glutamine, 100 units/ml of penicillin, and 0.1 mg/ml of streptomycin. The three cell lines differentiate upon RA treatment [39–41]. Cell differentiation was induced by adding 10 μM all-trans retinoic acid (RA) (Sigma) to the cultures, followed by 10 days incubation (media was changed after 5 days).

### mRNA isolation and RT-qPCR

RT-qPCR was performed using RNA from SH-SY5Y, SMS-KCNR, and IMR-32 cells treated or not with RA, using the IllustraRNAspin mini purification kit (GE Healthcare Life Sciences). 1 μg of total RNA was reverse transcribed using RevertAidTM reverse transcriptase, oligo (dT)18 primers, and RiboLock and RNase inhibitor (all from Fermentas). qPCR was performed as previously described [42] using validated primer sets (Qiagen) specific for the classical PTPs and reference gene (hypoxanthine phosphoribosyltransferase 1 [HPRT1]). All quantifications were normalized to the HPRT1 reference gene data. Relative quantification was performed using the comparative ΔΔCt method. Significant up-regulation or down-regulation was considered with a threshold of 2 or − 2 fold change (Log_2_ scale), respectively. Non-significant up-regulation or down-regulation was considered between 1 and 2 fold change or between − 1 and − 2 fold change, respectively. Those classical PTPs displaying coordinated significant increases in the three cell lines were considered as PTPs with significant up-regulation, whereas those PTPs displaying coordinated significant changes in two cell lines and non-significant changes in one cell line were considered as PTPs with significant up-regulation or down-regulation trends.

### PTPN1 knock-down, MTS cell proliferation and immunoblot assays

PTPN1 knock-down was performed by transfection of specific siRNAs using Lipofectamine 3000 (Thermo Fisher) or PepMute (SignaGen) reagents following manufacturer’s protocol. PTPN1 siRNAs (siPTPN1 #1, SI00043827; siPTPN1 #2, SI00043806) were from Qiagen, and siNS (non-specific) and siGAPDH siRNAs were from Ambion. PTPN1 knock-down was verified 72 h post-transfection at mRNA level by isolation of RNA and RT-qPCR as described above. Cell proliferation was analyzed 72 h post-transfection using CellTiter 96® AQueous One Solution Cell Proliferation Assay (MTS) (Promega) following manufacturer’s protocol. For immunoblot analysis, SH-SY5Y cells were incubated in the presence of epidermal growth factor (EGF; 50 ng/ml) for 5 min, and whole cell protein extracts were prepared by cell lysis in ice-cold M-PER™ lysis buffer (ThermoFisher Scientific) supplemented with PhosSTOP phosphatase inhibitor and cOmplete protease inhibitor cocktails (Roche, Switzerland), followed by centrifugation at 15200 g for 10 min and collection of the supernatant. Proteins (50 μg) were resolved in 10% SDS-PAGE under reducing conditions and transferred to PVDF membranes (Merck Millipore). Immunoblotting was performed using anti-phosphotyrosine 4G10 (1/1000, 05–321, Merck Millipore) and anti-GAPDH (1/1000, sc-32,233, Santa Cruz Biotechnology) antibodies, followed by IRDye secondary antibody (LI-COR) and visualization by Odyssey® CLx Imaging System. Electrophoretic bands were quantified using ImageJ software.

### Statistical analysis of immunohistochemistry data

Statistical analysis was performed using IBM SPSS Statistics package, and we performed crosstabs Pearson Chi square analysis, using 2-sided asymptotic Significance to calculate *p*-values, and interval by interval symmetric measures for Pearson’s R value.

## Results

### PTPN1 knock-down and growth of NB cells

**Fig. 1:**
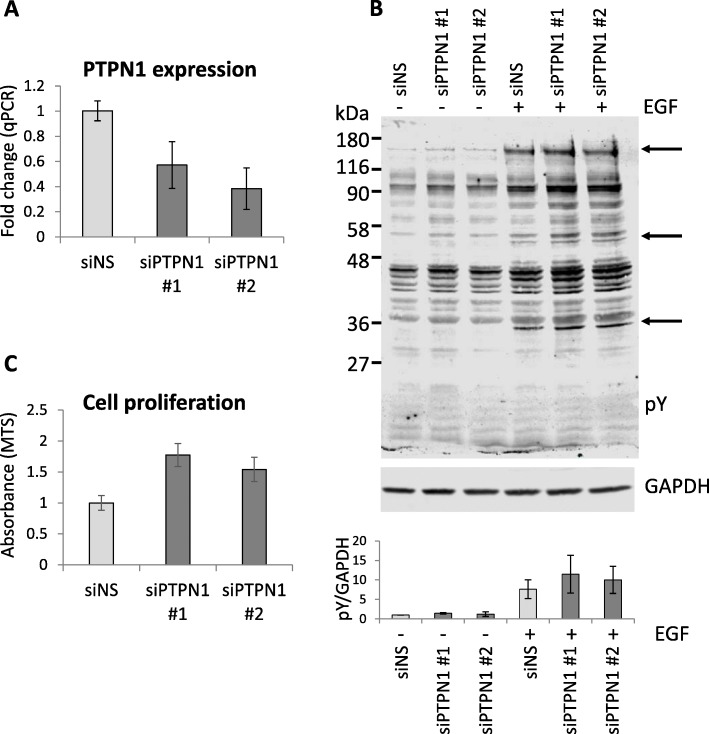
Tyrosine (Tyr) phosphorylation and proliferation of SH-SY5Y cells upon PTPN1 siRNA knock-down. Cells were transfected with control (siNS, non-specific) siRNAs or with two different siRNAs targeting PTPN1 (siPTPN1 #1, siPTPN1 #2), and assays were performed 72 h after transfection. **a** The silencing efficiency of the distinct siRNAs is shown, as monitored by RT-qPCR using specific primers. **b** Phosphotyrosine cell content was analysed by immunoblot with anti-phosphotyrosine 4G10 mAb (pY). GAPDH immunoblot was run as loading control. In the upper panel, a representative experiment is shown. In the lower panel, the quantification of phosphotyrosine content from each condition is shown. Results are shown as phosphotyrosine content normalized to GAPDH content (pY/GAPDH). **c** MTS cell proliferation; AU, arbitrary units. Data are shown as the mean + SD from two technical replicates from a representative experiment (**a** and **c**) or as the mean + SD from two independent experiments (**b**)

To investigate the impact of the expression of PTPN1 in the modulation of growth of human NB cells, SH-SY5Y cells were transiently transfected with specific siRNAs targeting PTPN1 for down-regulation (Fig. 1a), and phosphotyrosine content and MTS cell proliferation assays were performed 72 h after transfection. As shown, EGF short-term stimulation triggered tyrosine phosphorylation in SH-SY5Y cells, which was enhanced upon PTPN1 silencing, when compared to non-specific silencing conditions (Fig. 1b). Accordingly, siRNA down-regulation of PTPN1 expression resulted in increased cell proliferation (Fig. 1c). These results suggest a linkage between tyrosine phosphorylation status and proliferation of SH-SY5Y cells and illustrate the involvement of PTPN1 in the regulation of cell signaling and growth in NB.

### PTPN1 expression in human NB cells and NB tumor samples

**Fig. 2:**
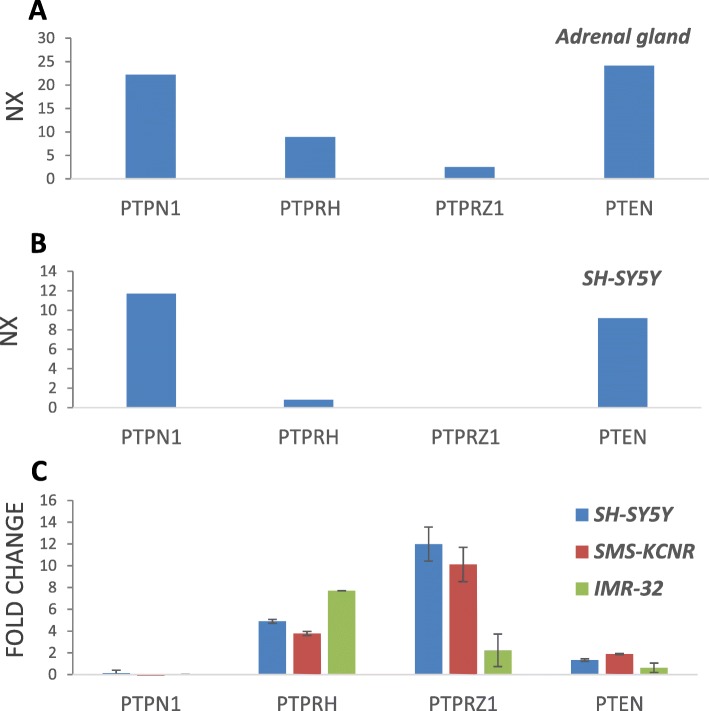
**a** mRNA expression of PTPs in Adrenal gland. Data from GTEx (Genotype-Tissue Expression) data sets. **b** mRNA expression of PTPs in SH-SY5Y cells. Data from HPA (Human Protein Atlas). Data in **a**. and **b**. are reported as transcription expression NX values (Normalized eXpression data, and is based on RNA-seqdata), according to https://www.proteinatlas.org/. c mRNA expression analysis of PTPs from human NB cell lines treated with RA. Cell lines were kept untreated or were treated for 10 days with RA, mRNA was extracted and RT-qPCR was performed using specific PTP primers. Relative mRNA expression values are shown in Log_2_ as fold change + S.D. of treated cells versus untreated cells, from at least two independent experiments. Mean fold change above 2 or below −2 was considered significant

The expression patterns of PTPN1 in human NB cells and NB tumor samples was investigated, in comparison with other PTPs, including PTPRH, PTPRZ1, and PTEN. The mRNA expression of these PTPs in the human adrenal gland (the major neuroendocrine tissue source of NB) and in the SH-SY5Y human NB cell line (the more studied human NB cell line) is shown in Fig. 2a and b, respectively. PTPN1 mRNA is abundantly detected from both of these sources, whereas other PTP mRNAs, such as PTPRZ1, are not detected. We also explored the potential role of these PTPs in NB differentiation, performing a quantitative RT-PCR (RT-qPCR) analysis of their mRNA expression on human NB cell lines differentiated in the presence of all-trans RA. These included SH-SY5Y, SMS-KCNR, and IMR-32 cell lines, which show different genetic backgrounds in terms of ALK mutational status and *MYCN* amplification. The cell lines were grown in the absence or in the presence of RA for 10 days, and mRNA was isolated and subjected to RT-qPCR using specific PTP oligonucleotide primers [42]. Figure 2c shows the relative changes of PTP mRNA expression from RA-treated cells in comparison with untreated cells. PTPN1 expression was not altered upon RA cell treatment, whereas the expression of PTPRH and PTPRZ1 was significantly up-regulated, and that of PTEN slightly up-regulated, in the three NB cell lines analysed.

**Fig. 3:**
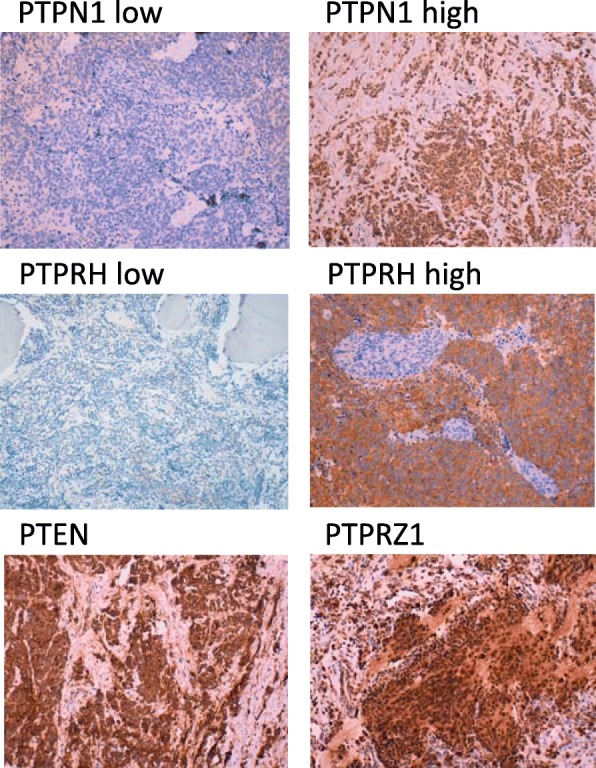
Immunostaining of PTPs from NB tissue sections. Representative PTPN1, PTPRH, PTPRZ1, and PTEN immunostaining patterns are shown. In the case of PTPN1 and PTPRH, selected images of low and high immunoreactivity are shown (magnification: X100)

Next, the expression on human NB tumor samples of PTPN1, PTPRH, PTPRZ1, and PTEN was analyzed by IHC using specific antibodies. A summary of our IHC results is shown in Table 2, and illustrative NB immunostained sections are shown in Fig. 3**.** PTPRH and PTPRZ1 were expressed in a large number of NB tumor samples, mostly displaying a cytoplasmic granular staining pattern. PTPRZ1 was expressed with high intensity in most of the tumor samples, suggesting that it could be a good marker in NB. High staining of PTPRH associated with low risk and non-IV stage. PTEN tumor suppressor was expressed in the majority of NB tumor samples and no clinical correlations were found, in agreement with previous reports [43], indicating that the lack of PTEN is not a common driving event in NB [44]. PTPN1 was also positive or weakly positive in most of the cases, with a cytoplasmic granular immunostaining. Importantly, high PTPN1 expression correlated with poor outcome parameters, including intermediate/high risk, metastasis and stages III/IV (Table 2). Further (Chi square) analysis revealed more significant correlations with low risk (*p* = 0.001, *R* = -0.501), non-metastatic (*p* = 0.042, *R* = -0.306), non-IV stage (*p* = 0.003, *R* = -0.448), and non-III/non-IV stage (*p* = 0.005, *R* = -0.420) for samples displaying high PTPRH and low PTPN1 expression. In conclusion, our findings suggest a differential involvement of PTPN1 and PTPRH in malignant NB progression, and argue for PTPN1 high expression as a potential surrogate marker for poor outcome NB.

**Table 2:** Immunostaining of PTPs from NB tissue sections

Patients (*n* = 44)	%	PTPN1		PTPRH		PTPRZ1		PTEN	
		high	low/no	high	low/no	high	low/no	high	low/no
Gender		(*p* = 0.3179) (*R* = 0.155)		(*p* = 0.571) (*R* = 0.068)		(*p* = 0.832) (*R* = 0.032)		(*p* = 0.4475) (*R* = 0.115)	
Male (21)	48	8	11	17	3	17	3	19	2
Female (23)	52	6	16	18	5	19	4	19	4
Age at diagnosis (months)		(*p* = 0.691) (*R* = 0.62)		(*p* = 0.243) (*R* = -0.178)		(*p* = 0.525) (*R* = -0.097)		(*p* = 0.376) (*R* = -0.133)	
< 18 (29)	66	9	19	25	4	25	4	26	3
> 18 (15)	34	8	5	10	4	11	3	12	3
MYCN		(*p* = 0.2551) (*R* = 0.204)		(*p* = 0.1036) (*R* = 0.283)		(*p* = 0.3719) (*R* = -0.155)		(*p* = 0.9699) (*R* = -0.007)	
Amplified (8)	18	4	3	5	3	6	0	7	1
Non-amplified (25)	57	8	16	22	3	22	3	22	3
Risk		**(*p*** **= 0.013)** (*R* = 0.394)		**(*p*** **= 0.023)** (*R* = -0.348)		(*p* = 0.241) (*R* = -0.179)		(*p* = 0.448) (*R* = -0.115)	
Intermediate, high(23)	52	10	11	15	7	17	5	19	4
Low (21)	48	3	17	20	1	2	19	19	2
Stage		**(*p*** **= 0.047)** (*R* = 0.310)		(*p* = 0.079) (*R* = 0.268)		(*p* = 0.335) (*R* = 0.147)		(*p* = 0.530) (*R* = 0.095)	
Metastatic (12)	27	6	4	7	4	9	3	11	1
Non-metastatic (32)	73	8	23	28	4	27	4	27	5
Stage		**(*p*** **= 0.008)** (*R* = 0.414)		(*p* = 0.101) (*R* = .250)		(*p* = 0.735) (*R* = -0.052)		(*p* = 0.234) (*R* = 0.179)	
III, IV (17)	39	9	6	11	5	13	3	16	1
non-III, non-IV (27)	61	5	21	24	3	23	4	22	5
Stage		(*p* = 0.095) (*R* = 0.260)		**(*p*** **= 0.001)** (*R* = -0.502)		(*p* = 0.059) (*R* = -0.287)		(*p* = 0.827) (*R* = -0.033)	
IV (13)	30	6	5	6	6	8	4	11	2
non-IV (31)	70	8	22	29	2	28	3	27	4
Survival		(*p* = 0.824) (*R* = 0.159)		(*p* = 0.2016) (*R* = -0.195)		(*p* = 0.1191) (*R* = -0.238)		(*p* = 0.505) (*R* = -0.101)	
Dead (10)	23	3	5	6	3	6	3	8	2
Alive (34)	77	11	22	29	5	30	4	30	4

PTP immunostaining was not obtained in some cases due to lack of tissue to perform the analysis or to non-informative immunostaining results

Significant *p* values (*p* < 0.05) are in bold

## Discussion

The efficacy of high-risk NB novel targeted therapies relies on a precise patient stratification based on novel genetic, epigenetic, or protein biomarkers [10, 45, 46]. In our study, we have found association of PTPN1 high expression with NB poor outcome, as indicated by PTPN1 enrichment in metastatic disease, and in samples from high stage and intermediate/high-risk NB patients. Interestingly, PTPRH expression displayed inverse clinical correlations than PTPN1 expression, which were increased when PTPRH high and PTPN1 low cases were clustered. This suggests PTPRH+/PTPN1- immunostaining of NB tumors as a good molecular predictor of low risk NB. Leptin or insulin treatment induced PTPN1 mRNA expression in SH-SY5Y cells [47]. However, PTPN1 mRNA expression did not display significant changes upon RA-induced differentiation of human NB cells (this report). On the other hand, high expression of PTPRH and PTPRZ1, which were coordinately up-regulated by RA in NB cell lines, was detected in our set of NB tumors. Interestingly, up-regulation of PTPRH has also been detected in breast cancer cell lines grown upon differentiation conditions [48]. How these PTP expression patterns in NB cells could be related with the differential expression and function of PTPs in NB tumors deserves further investigation. Noticeably, siRNA knock-down of PTPN1 resulted in increased tyrosine phosphorylation and cell proliferation of SH-SY5Y cells, indicating that PTPN1 could be important in regulating NB cell growth. This is in agreement with the report from Ozek et al. showing that PTPN1 inhibition increased the tyrosine phosphorylation and downstream signaling of TrkB, as well as the neurite outgrowth, in SH-SY5Y cells [49]. In addition, PTPN1 has been proposed to dephosphorylate the intracellular pools of ALK in mouse fibroblasts [50]. Together, our findings suggest that PTPN1 high expression in NB tumors might not be necessary for NB cell transformation, but rather could arise as a surrogate marker of NB tumor evolution, and support the hypothesis that PTPN1 growth-modulatory activity in SH-SY5Y NB cells takes place upstream in tyrosine phosphorylation signaling pathways. Further work is granted to understand the involvement of PTPN1 in NB cells growth and to validate PTPN1 as a novel NB prognostic biomarker.

## Conclusion

PTPN1 high expression in NB tumors associated with patient poor outcome, and PTPN1 knock-down affected the tyrosine phosphorylation status and proliferation of NB cells. This makes PTPN1 a suitable candidate for NB prognostic and intervention marker.

## Data Availability

All data generated or analysed during this study are included in this published article.

## Abbreviations

IHC: Immunohistochemistry
NB: Neuroblastoma
PTK: Protein tyrosine kinase
PTP: Protein tyrosine phosphatase
RA: Retinoic acid
RTK: Receptor tyrosine kinase
TMA: Tissue microarray
